# Progress in Anæsthetics

**Published:** 1900-10-13

**Authors:** 


### Progress in Anæsthetics.

**Ethyl Chloride.**—Wilsner[12] has given a prolonged trial to this anæsthetic and mentions the following points. The induction period is one or two minutes; there is little excitement, no respiratory or circulatory disturbance, and recovery is rapid without after effects. Muscular relaxation is, however, imperfect, and the patient is apt to recover consciousness during the operation. Its use is indicated in cases with circulatory or respiratory disorders, and in short operations. These views are borne out by Lotheissen,[13] who employs a modification of Clover's ether inhaler, and with 3 to 5 grammes of the drug secures an anæsthesia of from three to four minutes, so that it should be useful for dental operations.

The anæsthetic properties of ethyl bromide closely resemble those of the chloride. According to Larisch excitement is only seen in 8 per cent. of the adult cases and in less than 2 per cent. is there vomiting. The reflexes are never entirely abolished, but muscular relaxation is obtained. It should not be forgotten, however, that some years ago many accidents were reported from the use of this drug.

**Accidents during Anæsthesia.**—It is commonly alleged that chloroform may be given with perfect safety during parturition, but the comparative immunity from accident at this period is probably due to the fact that the quantity given is small, the anæsthesia never profound, and there is an absence of excitement or fear. Kidd, some years ago, detailed several cases of death from chloroform during childbirth, and a case where death followed the administration of 2 drachms is reported in the *British Medical Journal*, January 6th, 1900, page 35. Without going into details, we may mention several fatal cases of chloroform poisoning recently reported. Berry[14] records the case of a man to whom chloroform had recently been administered on two occasions, who on the third occasion died suddenly after the administration of 1 drachm by the open method. The death was from syncope. Atkinson[15] reports a similar death in a woman. In this case Krohne and Sesemann's regulating inhaler was used. Anæsthesia was obtained in 3 minutes, and in the whole time less than a drachm of chloroform was used. Bell,[16] at the same hospital (Hong Kong), had a fatal case in a man. Four drachms were given in 4½ minutes. A Skinner's inhaler was used. Cardiac failure was the mode of death in both cases. A series of fatalities under chloroform is detailed in the *Lancet*, June 17th, 1899. One of these, in which death was due to suffocation consequent upon the presence of vomited matter in the bronchi, illustrates very forcibly the necessity of washing out the stomach before all emergency operations.

Wilson[17] points out the dangers that may arise from the administration of chloroform in confined rooms with naked lights burning. The vapour is decomposed, forming carbonyl chloride (phosgene gas), which at first, exciting irritation in the air passages, may eventually lead to pneumonia and œdema of the lung. In this way cases are on record[18] in which patients, doctors, and nurses have succumbed. Free ventilation of the operating room is essential.

Sickness follows the administration of an anæsthetic in about two-thirds of the cases. Blumfield[19] thinks that persons habitually using alcohol and tobacco are less likely to vomit than others, although much depends on idiosyncrasy. Patients who secrete much mucus during the operation are generally sick, and it is advisable sometimes to wash out the stomach with warm water before the patient comes round. The longer the administration the greater the risk of after-sickness. Vomiting is also likely to follow operations in which blood is swallowed, e.g. adenoids, and is also more common if the patient be moved whilst under the influence of the drug. To prevent after-sickness nothing except sips of hot water, or of tea or coffee, should be allowed for at least eight hours; the room should be kept warm. Brandy and soda water, iced champagne, or sniffing at strong sal-volatile will frequently check vomiting. Two cases of jaundice following chloroform administration are recorded by Brainerd.[20] This is a very rare sequela.

Localised paralysis is not an uncommon condition following anæsthesia. Mally[21] has investigated 36 such cases and finds that 10 were of central origin, 6 hysterical, 17 peripheral, and 3 reflex. The hysterical and reflex paralysis he regards as being free from all connection with the anæsthesia; in the central cases the bursting of a vessel may depend to a great extent on struggling movements during the stage of excitement; the peripheral lesions were due to accidental compression of nerve trunks or the roots of the brachial plexus in the course of the operation. To guard against this last danger the patient's arms should not be dragged up to the head, permitted to hang over the edge of the table, or lie under the trunk during anæsthesia. There is no evidence to prove that chloroform or ether has a direct toxic paralysing effect on nerve trunks or endings. The Society of Anæsthetists,[22] who recently discussed this subject, arrived at identical conclusions. There is, however, some difficulty in accounting for post-anæsthic facial paralysis. Low, in the course of the discussion, mentioned the case of a woman who had a series of epileptiform fits whilst under ether and oxygen. There was no previous history of epilepsy. Turney suggested that the cause might be a small focal hæmorrhage, and Paterson that the convulsions were due to an excess of oxygen. Savage,[23] in an interesting lecture on the relationships between anæsthetics and insanity, states that in predisposed subjects, in neurotic and degenerating states, an anæsthetic may be the exciting cause of an attack of mania or stupor. Either chloroform or ether may be given to the insane with impunity if operations are necessary, and may relieve for a time a few maniacal cases. If given to persons who have previously had attacks of insanity there is a danger that it may lead to a fresh outbreak, and it frequently does so in persons who suffer from recurrent attacks. He gives cases of acute mania following gas administration and of mania following the administration of chloroform in childbirth.

**Local Anæsthesia.**—The infiltration method has met with considerable success at the hands of Barker.[24] He uses a saline solution with 1 in 1,000 of eucaine B. added to it. As much as 5 or 6 ounces may be used for one operation, and no ill effects beyond slight vomiting and pallor have been witnessed. The syringe and needle should be sterilised, and the solution injected warm. The effect of the drug is complete in 5 minutes, and fresh material should be injected in 20 minutes. The method should not be employed where rapidity of operation is essential; it is unsuited for inflamed tissues, for children, or timid adults. Amongst the operations performed by Barker under this method are radical cure for hernia (15 times), removal of goitre, colotomy, empyema, &c. Lilienthal[25] has injected as much as 4 to 10 grains of eucaine at one operation. He first injects a small dose of morphia, then anæsthetises the skin, and when the deeper structures are reached injects the nerve itself. Chloroform may follow eucaine if necessary. Irzebicky[23] substitutes for eucaine one of Schleich's solutions—cocaine hydrochlor. ·1, morphine hydrochlor. ·02, sod. chlor. ·2, aqua 100. In only 5 out of 181 operations did pain necessitate the use of chloroform. The average amount used was 30 to 40 cubic centimetres. The whole field of operations should be rendered œdematous by the injections, especial attention being paid to the skin. The average period of anæsthesia was one hour. Stone[27] also reports favourably on Schleich's solutions. Bier's[28] method of injecting with cocaine the tissues over the lumbar spine and the sac of the spinal cord, and so producing anæsthesia below the seat of puncture, seems open to many and grave objections. The repeated punctures necessary in the infiltration processes have been an objection. Milian[29] employs a 5 per cent. solution of cocaine in chloride of ethyl. This is either applied on a pledget of cotton wool or from an atomiser. The chloride of ethyl, removing the fats from the skin, enables the cocaine to penetrate. Anæsthesia is obtained in about 5 minutes.

[12] Wien. Med. Woch., July 8, 1899. [13] Arch. F. Kl. Med., 57, 4. [14] Lancet, Dec. 30, 1899. [15] Lancet, Nov. 4, 1899. [16] Ibid. [17] Bost. Med. Chi. Jour., May 11, 1899. [18] Lancet, June 24, 1899. [19] Lancet, Sept. 23, 1899. [20] Univ. Med. Mag., May, 1899. [21] Rev. de Chir., July, 1899. [22] Clin. Jour., July 12, 26, 1899. [23] Clin. Jour., Apr. 11, 1899. [24] Lancet, June 20, 1900. [25] Med. Rec., June 10, 1899. [26] Wien. Med. Woch., No. 13, 1899. [27] Jour. Am. Med. Assoc., No. 4, 1899. [28] Deut. Leit. f. Chir., vol. li., page 3. [29] Rev. Méd., May 24, 1899.

